# Evaluation of Sexual Harassment Policies at Medical Institutions to Understand Attention to Harassment of Physicians by Patients

**DOI:** 10.1001/jamanetworkopen.2021.35131

**Published:** 2021-11-17

**Authors:** Gabriela Reed, Sarah R. Ahmad, Elaine C. Khoong, Kristan Olazo, Reshma Jagsi, Christina Mangurian, Urmimala Sarkar

**Affiliations:** 1 Department of Internal Medicine, University of California, San Francisco; 2 Division of Headache Medicine, Department of Neurology, University of California, San Francisco; 3 Division of General Internal Medicine, University of California, San Francisco; 4 Center for Vulnerable Populations, University of California, San Francisco; 5 Department of Radiation Oncology, University of Michigan, Ann Arbor; 6 Department of Psychiatry and Behavioral Sciences, University of California, San Francisco

## Abstract

This quality improvement study assesses the policies of top US medical centers for addressing sexual harassment of physicians by patients.

## Introduction

Sexual harassment in medicine negatively impacts professional and psychological well-being while creating an unsafe work environment.^1,2^ An understudied type of sexual harassment is harassment of physicians by patients and/or patient family members.^1^ We evaluated whether top medical centers had policies that addressed sexual harassment of physicians by patients.

## Methods

We used the Standards for Quality Improvement Reporting Excellence (SQUIRE) reporting guidelines for quality improvement studies.^3^ This study was exempt from review by the University of California, San Francisco, institutional review board. We identified the top 50 US hospitals using 2020 *Newsweek* rankings, which represent a geographically diverse group of academic and community hospitals.^4^ We conducted an online search of publicly available documents and determined that the internal, employee-centered policies we sought were not readily available.

Between September and November 2020, we emailed the chief medical officers (or equivalent), requesting their policies on sexual harassment by patients and/or their families toward physicians. Institutions were contacted up to 3 times. If institutions were unable to share documents, we sent follow-up emails requesting policy details.

Two investigators (G.E.R. and S.R.A.) used deductive coding of all documents and emails. In cases of disagreement, the study team met to reach consensus. Any policy reference to “staff” or a similar descriptor (eg, “provider,” “faculty,” “employee”) was assumed to apply to physicians. We categorized policies as sexual harassment, inappropriate behavior, patient rights/responsibilities, employee workplace safety, or other patient-facing material. We extracted whether the policy explicitly mentioned any of the following behaviors: sexual harassment, physical assault/violence, verbal harassment/intimidation, or discrimination. We used the standard definition of discrimination as differential negative treatment based on race, gender, sexual orientation, ability, or other protected category. We coded whether policies addressed the harassment of health care staff by patients or family specifically. We noted any description of institutional response. For single documents applicable to multiple hospitals in one system, the document was counted once for each institution.

## Results

Of 50 institutions, 28 (56%) responded (Figure). Four institutions reported no applicable policies, 6 reported having a policy but were unable to share, and 18 shared one or more policies or relevant documents. Six institutions not sharing their policy provided a description by email. We characterized policies at 24 institutions (Table). This process yielded a total of 38 unique documents: 8 sexual harassment policies, 14 inappropriate behavior policies, 7 patient rights/responsibilities policies, 8 employee workplace safety documents, and 1 flyer. The following behaviors were explicitly described in at least 1 policy: sexual harassment (18 of 24 [75%]), physical assault/ violence (14 of 24 [58%]), verbal harassment/intimidation (12 of 24 [50%]), and discrimination (13 of 24 [54%]). Seventeen institutions described a response to inappropriate behavior, one of which included a designated response team to support those who experienced harassment. Of the 24 institutions that endorsed having applicable policies, 14 (59%) addressed sexual harassment from patients toward physicians. Seven policies, applicable to 11 institutions, mentioned patients among lists of potentially involved parties that included broad groups such as employees, volunteers, and visitors. Only 1 policy, in 3 institutions, specifically mentioned sexual harassment with regard to patient treatment of staff.

**Figure.**
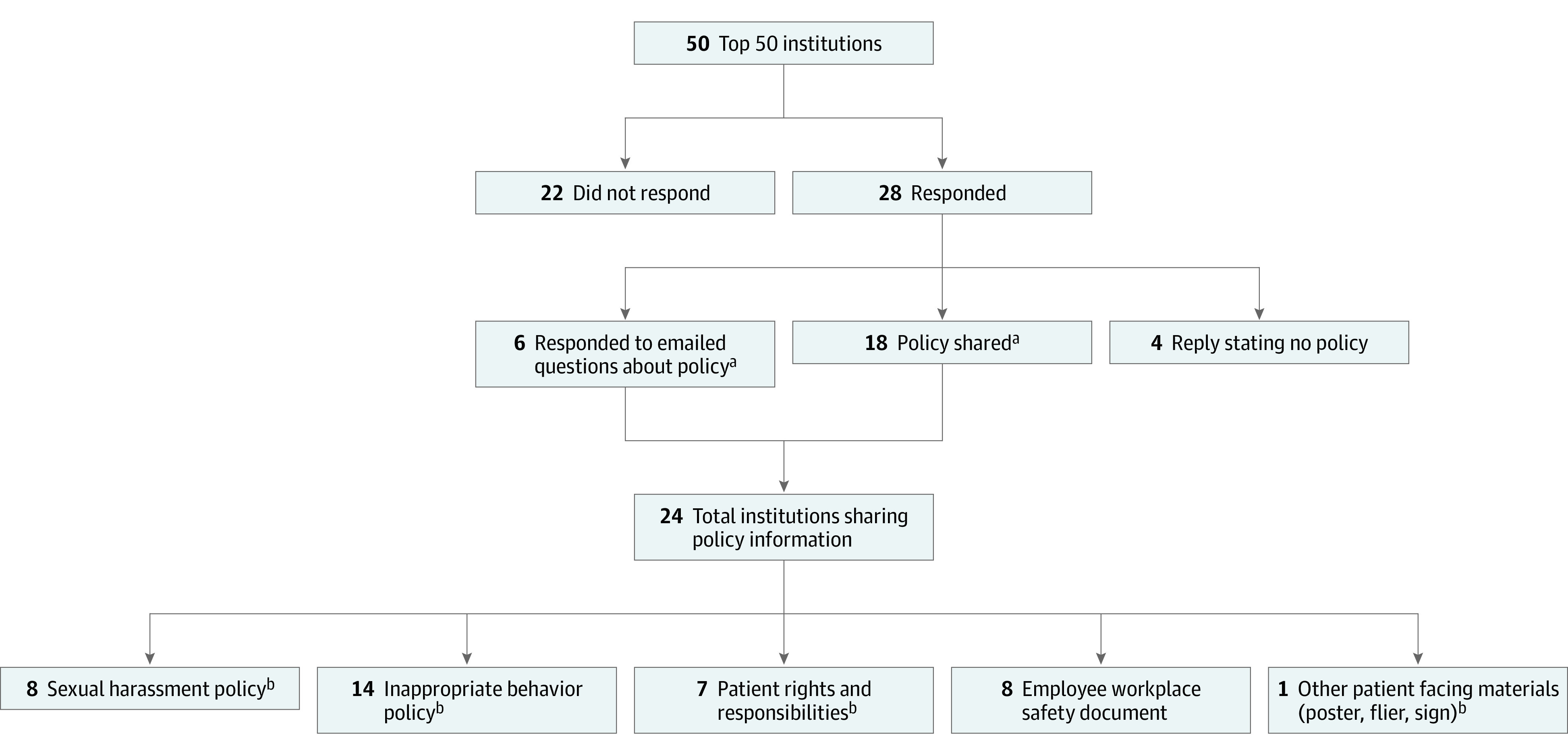
Hospitals Included in the Study Study flowchart of institutional policy information included. This flowchart shows the number of institutions identified and contacted from the top 50 US hospitals using the 2020 *Newsweek* rankings.^3^ Twenty-four policy documents or emails were included in the analysis. ^a^Some institutions were covered by a joint policy and were considered as having shared their policy. ^b^Some institutions shared more than one type of policy.

**Table.** Characteristics of Policies Addressing Inappropriate Behavior, by Institution

Institutional policy characteristics (N = 24 institutions)	No. (%)
Forms of inappropriate behavior specifically mentioned^a^	
Sexual harassment^b^	18 (75)
Physical assault/violence	14 (58)
Verbal harassment/intimidation	12 (50)
Discrimination	13 (54)
Specifically addresses inappropriate behavior from patients toward physicians^c^	
Yes, specific mention of how patients are expected to treat all health care staff	13 (54)
Yes, patients mentioned as part of a list of potential perpetrators	12 (50)
Specifically addresses sexual harassment from patients toward physicians	
Yes, specific mention of how patients are expected to treat all health care staff	3 (13)
Yes, patients mentioned as part of a list of potential perpetrators	11 (46)
Describes institutional response to inappropriate behavior	
Yes	17 (71)
Describes the time frame of an institutional response	
Yes	7 (29)

^a^ Some institutions shared more than one type of document.

^b^ National Academies of Sciences, Engineering, and Medicine’s definition of sexual harassment was used.^1^

^c^ Some institutions provided more than one document with specific mention of how patients are expected to treat staff and as part of a list of potential perpetrators.

## Discussion

We found a dearth of policies that specifically address sexual harassment from patients toward physicians. This study of internal, non–public facing policies and documents aligns with prior analyses showing publicly available patient rights/responsibilities policies rarely address sexual harassment.^5^ Communication with chief medical officers revealed a lack of standardization, as demonstrated by the wide array of document types received, variable and limited information provided, and differences in approach when such behavior occurs. Institutions can rely on exemplar policies like Mayo Clinic’s, which clearly defines sexual harassment and outlines a stepwise institutional response.^6^ The physician workforce continues to diversify, and women now outnumber men among younger physicians. Women, especially women who belong to racial and/or ethnic minority groups, experience more harassment than their male counterparts.^1^ While this study is limited by response and selection bias, we hope that highlighting top hospitals’ policies may galvanize constructive changes to other institutional policies.
